# Too Many Treats or Not Enough to Eat? The Impact of Caregiving Grandparents on Child Food Security and Nutrition

**DOI:** 10.3390/ijerph19105796

**Published:** 2022-05-10

**Authors:** Rahel Mathews, Danielle Nadorff

**Affiliations:** 1 Department of Food Science, Nutrition and Health Promotion, Mississippi State University, Starkville, MS 39762, USA; 2 Department of Psychology, Mississippi State University, Starkville, MS 39762, USA; danielle.nadorff@msstate.edu

**Keywords:** grandchildren, grandparents, BMI, nutrition, food security, obesity, children, adolescents, hunger

## Abstract

With the number of grandparent-headed households on the rise, the influence of grandparents needs to be considered in the fight to reduce child obesity. The current study investigated the influence of caregiver type (i.e., grandparents only, parents only, or multi-generational households) on children’s nutrition, food security, and BMI. This was a cross-sectional, secondary analysis based on the 2009–2010 wave of the Health Behavior in School-Aged Children (HBSC) survey in collaboration with the World Health Organization. This sample included 12,181 students from 10,837 families with only parents present in the household, 238 with only grandparents present, and 1106 multi-generational families. One-way analyses of covariance (ANCOVAs) were conducted using caregiver type as the independent variable, controlling for SES, on items assessing frequency of breakfast consumption, nutrition intake, hunger, snacking frequency and location, and BMI. Children reported more unhealthy snacking in households with only grandparents. Hunger was reported more often in multi-generational households. These results support that caregiver type, especially caregiving grandparents, is a significant predictor of children’s BMI, nutrition, and food security. Tailoring nutrition education to the needs of grandparents could help both the health of grandparents and the reduction of child obesity.

## 1. Introduction

The US national prevalence of obesity for children is an alarming 17% [1], making it one of the primary public health burdens in the US. A growing number of children are now experiencing chronic health problems such as type II diabetes, hypertension, and other adult-onset adverse health outcomes, even during childhood. Moreover, childhood obesity may be associated with an increased likelihood of adult obesity [2,3,4].

According to the socioecological model, a child’s weight status can be influenced by immediate as well as distal factors related to parenting style, family, and community [5,6]. The literature reflects a large emphasis focusing on children and their parents, mostly mothers. However, according to the 2016 US Census, 7.5 million grandchildren were living with their grandparents. Of these, roughly 2.5 million were skipped-generation households in which grandparents were solely responsible for meeting the needs of their grandchildren [7]. Children typically resided with their grandparents due to the fact of stressful or traumatic situations with the most common being divorce, parental drug abuse, and child abuse [8]. Research has shown that these families often face significant financial hardship [9,10]. This hardship is primarily because custodial grandparents are often too young to draw retirement benefits, and 85% do not receive any public assistance for themselves or their grandchildren [11]. Relatedly, 33% of custodial grandchildren lack health insurance [12,13]. As such, these grandparents and their grandchildren are at elevated risk for many health and social conditions [14] including obesity [15] and food insecurity [16].

As the literature currently stands, the role of grandparents has been examined in child nutrition, mostly in terms of grandparents helping as intermittent caretakers or as caregivers in a multi-generational household. A systematic review of the literature from 2000 to 2017 revealed 16 studies globally of which only 6 US studies pertained to grandparents and how they influence their grandchildren’s dietary behaviors [17]. These few studies report a variety of dietary behaviors that can affect children. Grandparents may provide a positive feeding environment, including role-modeling healthy food intake, teaching children about nutrition, involving them in mealtimes and cooking, monitoring, and encouraging children to eat nutritious foods [18], and regularly serving vegetables [19]. However, grandparents also reported providing energy-dense and nutrient-poor food and drinks and used food as a reward or gift [18,20], which were categorized as negatively influencing children [17]. Further, in multi-generational households, when grandmothers were present and/or prepared food, the odds of a higher child weight increased approximately 1.5 times across three studies—in Japan [21], China [22], and the US [23]—and four times in one study of Greek households [24]. In contrast, one study found that in some Hispanic cultures, children who had Hispanic grandmother caretakers had lower zBMI scores than those who did not have a grandparent involved [25].

The food environment at home may be influenced by other family and community factors [5]. When there was more disagreement between the parent and grandparent, more negative feeding practices were reported [17], and in one study, this conflict was associated with an increased child zBMI score [17,25]. Age, gender, and educational attainment of the grandparent may also influence dietary practices with children [26]. For example, in a study of over 1000 grandparents in Australia, the authors found that male grandparents, those residing in lower SES areas, and those with lower educational attainment were more likely to practice negative feeding habits, while female grandparents, those living in higher SES, and those of higher educational attainment were likely to report positive practices. Older grandparents in that same study were more likely to control what and when their grandchild ate but, at the same time, were less likely to offer praise for eating healthy foods [26].

Caregiving grandparents tend to “parent” differently when they have more time and charge of grandchildren [18]. In “skipped-generation” or households where grandparents have a primary responsibility for the care of the children without their parents present, grandparents may take an increased interest in serving healthier options, as the more hours grandparents spend with grandchildren, the more their feeding behaviors resemble those of parents [18].

Current literature documents the role of grandparents in multi-generational and skipped-generation households but does not sufficiently explore the impact of differential caregiver types (including skipped-generation grandparents) on children’s health. There is not enough quantitative data to characterize how caregiving grandparents can affect child BMI and childhood obesity. Understanding this data can help develop and customize programs to aid grandparents not only with their health but also that of their family.

The purpose of this study was to compare the dietary and physical activity behaviors in households where adolescents live with their grandparents. We compared homes with parents and grandparents (i.e., multi-generational) to homes headed by grandparents only (i.e., skipped generation) and with homes in which children lived only with their parents (i.e., parents only). **Based on previous literature, the following hypotheses were made**: 1. Adolescents living with grandparents (either skipped-generation or multi-generation) would report poorer nutrition behaviors than those living in parents-only households. 2. Adolescents living with grandparents (either skipped-generation or multi-generation) would report higher BMI scores than those living in parents-only households. 3. Adolescents living with grandparents (either skipped-generation or multi-generation) would report more food insecurity than those living with parents in the home.

## 2. Materials and Methods

Data were taken from the 2009–2010 wave of the Health Behavior in School-Aged Children (HBSC) survey, a series of international data collected in collaboration with the World Health Organization [27]. This dataset included 12,642 students in 314 schools nationwide from the United States. Details on the survey can be found at: http://www.hbsc.org (accessed on 29 April 2022). Briefly, the HBSC studies are cross-sectional studies repeated every 4 years on 11, 13, and 15 year old boys and girls, with the objectives of monitoring health-risk behaviors and attitudes and promoting healthy initiatives. Students were recruited from a nationally representative sample of public, private, and Catholic schools, with African American and Hispanic students being oversampled, in grades 5–10. Specifically, within our sample, participants ranged in age from 10 to 17 with a mean age of 12.95 (*SD* = 1.75).

### 2.1. Measures

Caregiver Type: Children in the sample were separated by caregiver type into three groups: Parents Only (those who lived with either a father or mother or both, but no grandparents); Grandparents Only (those who lived with a grandfather or grandmother or both, but no parents): Multi-Generational (those who lived in a home with at least one parent and one grandparent).

Nutrition Behaviors:

Frequency of Consuming Breakfast: Prior research has shown that consumption of breakfast before attending classes may be associated with better cognitive and academic performance [28]. The HBSC included two questions assessing how often these adolescents were eating breakfast:

How often do you usually have breakfast (more than a glass of milk or fruit juice) on weekdays? (six-point response scale ranging from 0 to 5 days);

How often do you usually have breakfast (more than a glass of milk or fruit juice) on weekends? (three-point response scale ranging from 0 to 2 days).

Nutrition Intake: Adolescents are known for their low vegetable and fruit intake and consumption of high caloric food and drinks [29]. Food intake may be indicative of developing life-long eating habits [4]; however, their nutritional intake can also be influenced by individual and environmental factors [5]. The validation and piloting of the food frequency items as well as the other items in the HBSC are discussed in their materials [27]. In brief, the food frequency items (nutrition intake in our study) were piloted and retested in several countries. Their validation process included comparing the results of these items to a 24 h recall and a 7 day food diary [30]. Five questions assessed participant intake of nutritious and non-nutritious food types:

How many times a week do you usually eat or drink fruits? (seven-point response scale ranging from “Never” to “Every day, more than once”);

How many times a week do you usually eat or drink vegetables? (seven-point response scale ranging from “Never” to “Every day, more than once”);

How many times a week do you usually eat or drink sweets (candy or chocolates)? (seven-point response scale ranging from “Never” to “Every day, more than once”);

How many times a week do you usually eat or drink Coke or other soft drinks that contain sugar? (seven-point response scale ranging from “Never” to “Every day, more than once”);

How often do you eat in a fast-food restaurant (for example, McDonalds, KFC, Pizza Hut, Taco Bell)? (seven-point response scale ranging from “Never” to “5 or more days a week”).

Snacking Frequency and Location: Adolescents consume a large portion of their calories from snacks. Additionally, snacking in front of the television or computer is sedentary behavior that is associated with higher caloric intake and weight gain [31,32]. Two questions assessed the frequency and location of snacking behaviors:

How often do you snack while you watch TV (including videos and DVDs)? (six-point response scale ranging from “Never” to “Every Day”);

How often do you snack while you work or play on a computer or games console? (six-point response scale ranging from “Never” to “Every Day”).

Hunger/Food Security: A single item was included assessing reported hunger: “Some young people go to school or bed hungry because there is not enough food at home. How often does this happen to you?” (four-point response scale ranging from 1 = “Always” to 4 = “Never”].

To describe the range of nutrition behaviors more fully in the population, each of the above questions were included within the analyses separately, without any scale creation.

Computed BMI Category: Body Mass Index (BMI) was based on the “BMI_Comp” variable within the HBSC dataset, which first calculates BMI using the following standardized formula: (weight (lbs)/(height (inches) × height (inches))) × 703. BMI Category is applied based on the children’s sex and age using the year 2000 Centers for Disease Control’s percentiles (found at: http://www.cdc.gov/growthcharts/clinical_charts.htm (accessed on 29 April 2022)) to create percentile categories ranging from 1 = “underweight” (less than the 5th percentile); 2 = “healthy weight” (between the 5th–85th percentile; 3 = “at risk of overweight” (between the 85th and 95th percentile); 4 = “overweight” (greater than the 95th percentile) [33].

SES: Socioeconomic status was based on the “family affluence scale” (FAS) [27], a four-item measure of self-reported familial wealth in adolescents. The FAS has been found to have good criterion validity and country-rank order correlation with the gross domestic product, making it a valid measure of SES [34].

### 2.2. Statistical Analysis Plan

One-way analyses of covariance (ANCOVAs) were conducted using caregiver type as the independent variable, controlling for SES, on each of the questions assessing frequency of breakfast consumption, nutrition intake, snacking frequency and location, food insecurity, and BMI. Missing data were addressed through the use of listwise deletion. Data are the adjusted mean ± standard error, unless otherwise stated. Analysis was conducted in SPSS version 26 for Mac. Chi-square analysis was used to compare the proportions of overweight adolescents by caregiver type.

## 3. Results

Within our sample of adolescents, 12,181 indicated they were from a house where they lived with only their parents (*n* = 10,837), only their grandparents (*n* = 238), or in a multi-generational home (*n* = 1106). Four hundred and sixty-one adolescents did not fit into our categories. Our study data were 51.1% male, and the mean age was 12.95 years (*SD* = 1.74). Approximately forty-seven percent (47.2%) of the sample was Caucasian, 16.8% African American, and 19% reported being of Hispanic ethnicity.

A summary of the descriptive statistics for the output measures are provided in Table 1. There was a significant effect of caregiver type on the frequency of eating breakfast on weekdays, controlling for SES: *F*(2, 11,902) = 4.149, *p* < 0.05, partial η^2^ = 0.001. Post hoc analysis was performed with a Bonferroni adjustment, but it did not reveal statistically individual significant group differences. Additionally, after controlling for SES, there was no significant effect of caregiver type on the frequency of eating breakfast on weekends.

Regarding nutrition intake, after controlling for SES, there was a significant effect of caregiver type on the frequency of consuming “sweets” each week: *F*(2, 11,294) = 6.333, *p* < 0.01, partial η^2^ = 0.001. Post hoc analysis was performed with a Bonferroni adjustment. Consumption of sweets was statistically significantly greater in the Grandparents-Only group versus the Parents-Only group (mean difference of 0.440 (95% CI, 4.507–4.067), *p* < 0.01) with no significant differences related to the Multi-Generational group (4.06 ± 1.855). Similarly, after controlling for SES, there was a significant effect of caregiver type on the frequency of drinking soft drinks each week: *F*(2, 11,421) = 13.759, *p* < 0.001, partial η^2^ = 0.002. Post hoc analysis was performed with a Bonferroni adjustment. Consumption of soft drinks was statistically significantly greater in the Grandparents-Only group versus the Parents-Only group (mean difference of 0.612 (95% CI, 4.724–4.112), *p* < 0.001) and the Multigenerational group (mean difference of 0.416 (95% CI, 4.724–4.308), *p* < 0.05). Additionally, the Multi-Generational group consumed a statistically significantly greater number of soft drinks than the Parents-Only group (mean difference of 0.196 (95% CI, 4.308–4.112), *p* < 0.001).

Similar results were found for the frequency of consuming fast food, with a significant effect of caregiver type, after controlling for SES: *F*(2, 11,895) = 8.855, *p* < 0.001, partial η^2^ = 0.001. Post hoc analysis was performed with a Bonferroni adjustment. Frequency of eating fast food was statistically significantly greater in the Grandparents-Only group versus the Parents-Only group (mean difference of 0.365 (95% CI, 4.245–3.881), *p* < 0.01) and the Multi-Generational group (mean difference of 0.265 (95% CI, 4.245–3.980), *p* < 0.05). However, after controlling for SES, there was no significant effect of caregiver type on how often children consumed fruits or vegetables each week.

**Figure 1:**
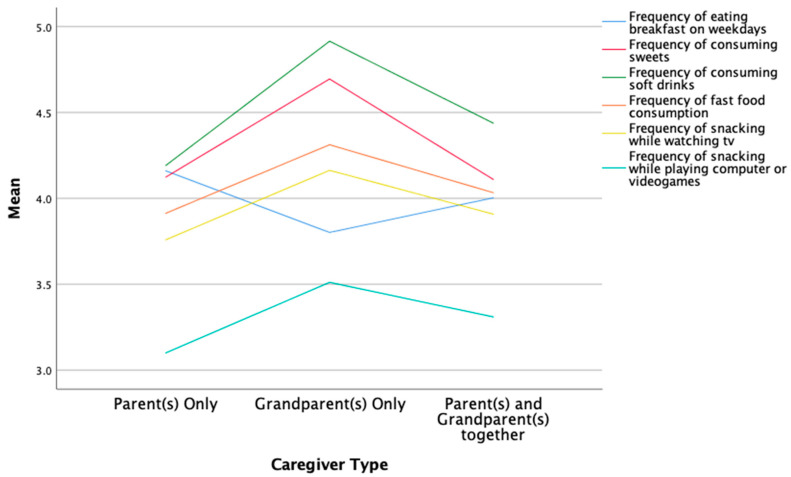
Significant group differences by caregiver type in the frequency of eating breakfast, nutritional intake, and snacking behavior.

Regarding snacking frequency and location, after controlling for SES, there was a significant effect of caregiver status on how often children snacked while watching tv: *F*(2, 8302) = 7.303, *p* < 0.01, partial η^2^ = 0.002. Post hoc analysis was performed with a Bonferroni adjustment. Snacking in front of the tv was statistically significantly more frequent in the Grandparents-Only group versus the Parents-Only group (mean difference of 0.430 (95% CI, 4.190–3.759), *p* < 0.01), with no differences related to the Multi-Generational group (3.90 ± 1.657). Similarly, after controlling for SES, there was a significant effect of caregiver status on the frequency of snacking behavior while using a computer or gaming system: *F*(2, 8233) = 7.349, *p* < 0.01, partial η^2^ = 0.002. Post hoc analysis was performed with a Bonferroni adjustment. Snacking while playing videogames was statistically significant in the Grandparents-Only group versus the Parents-Only group (mean difference of 0.406 (95% CI, 3.512–3.105), *p* < 0.01) and in the Multi-Generational versus the Parents-Only groups (mean difference of 0.191 (95% CI, 3.296–3.105), *p* < 0.01). See Figure 1 for a visual representation of the above findings.

**Figure 2:**
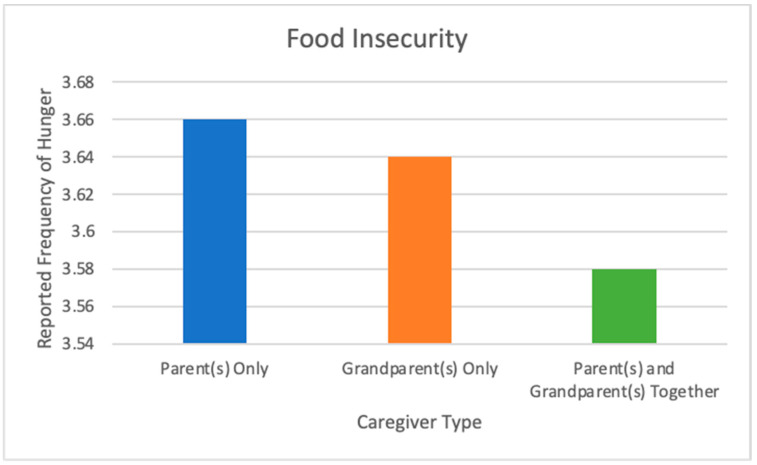
Adolescents’ reported frequency of hunger grouped by type of primary caregiver, controlling for socioeconomic status. Responses were measured regarding how often they went to school or bed feeling hungry because there was no food at home, where 1 was “always” and 4 was “never”. Higher bars indicate more food security and going hungry less often. Adolescents in multi-generational households were significantly more likely to report going hungry more often.

Regarding food insecurity, there was a significant effect of caregiver type, after controlling for SES, on the frequency of children going to school or bed hungry: *F*(2, 11,831) = 7.643, *p* < 0.001, partial η^2^ = 0.001 (See Figure 2). Post hoc analysis was performed with a Bonferroni adjustment. Food insecurity was statistically significant when comparing the Parents-Only group versus the Multi-Generational group (mean difference of 0.080 (95% CI, 3.659–3.579), *p* < 0.001), with no statistically significant differences related to the Grandparents-Only group (3.64 ± 0.691). Note that higher means indicate more food security and lower reports of going hungry.

**Figure 3:**
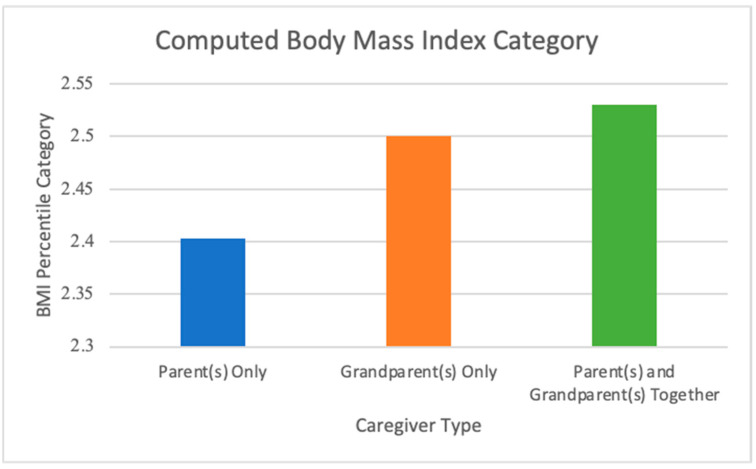
Adolescents’ average computed Body Mass Index percentile grouped by type of primary caregiver, controlling for socioeconomic status. Categories range from 1 = underweight (less than 5th percentile) to 4 = overweight (greater than 95th percentile) based on 2000 CDC categorizations.

Finally, after controlling for SES, there was also a significant effect of caregiver type on adolescents’ computed BMI percentile category: *F*(3, 9808) = 11.519, *p* < 0.001, partial η^2^ = 0.001 (See Figure 3). Post hoc analysis was performed with a Bonferroni adjustment. The average computed body mass index was statistically significantly higher in the Multigenerational group versus the Parents-Only group, (mean difference of 0.127 (95% CI, 0.60–0.193), *p* < 0.001), with no statistically significant differences related to the Grandparents-Only group (2.500 ± 0.026) (Figure 3). When comparing the proportion of overweight children by caregiver type (*n* = 9981), there were 1179 (13.3%) in the Parent(s)-Only group, 32 (18.1%) in the Grandparent(s)-Only group, and 152 (17.7%) within the Multi-Generational group. A chi-square test revealed that this was a statistically significant difference in proportions of overweight children (*p* < 0.001).

## 4. Discussion

Child obesity needs to be studied within many contexts. The influences of adults on a children’s diet and physical activity need to be considered beyond the parent–child dyad to that of extended kin, most importantly grandparents [5]. Using a nationally representative sample of adolescents, this was the first study of its kind to compare nutritional intake, food security, and BMI among children raised by their grandparents, those in a multi-generational home, and those raised solely by their parents. Our results show that grandparents do have an influence on children’s dietary intake and BMI.

The United States Department of Agriculture defines food insecurity in terms of the lack of access to safe and nutritious foods at all times [35]. In this study, food insecurity was defined by children reporting going to school or to bed hungry when there is no food at home. Additionally, food insecurity is also associated with a nutritionally poor diet and obesity [36], which were additional outcome variables in this study.

The first hypothesis was partially supported: after controlling for SES, those living with grandparents, especially those in skipped-generation households (grandparents only), reported significantly poorer nutritional intake than those living with parents in the areas of sweets, soft drinks, fast food, and snacking while watching tv and videogames. However, there were no significant effects of caregiver type on fruit or veggie consumption. This fits with the mixed findings of Young et al. [17], who found that grandparents are likely to both model healthy behaviors, such as serving vegetables, but also to serve their grandchildren nutrient-poor foods. While adults may have positive attitudes towards healthy foods, they may find it challenging to meet the costs of healthy food while also meeting the demands of young people who will not always appreciate the effort. Nutrition education can help grandparents gain confidence in serving healthy but low-cost snacks [37].

The second hypothesis was partially supported, with those living in multigenerational households having significantly higher BMI scores than those living with only parents. It is important to note that all three groups of adolescents (regardless of caregiver type) reported an average BMI percentile category between two and three, which corresponds to being of normal weight—at risk of overweight. We also found that the proportion of overweight children was significantly different between parents and grandparents. This substantiates the findings of previous literature [21,22,23,24,38], where children’s BMIs were higher in multi-generational families. Although there were no significant differences found for adolescents living in houses headed by grandparents only, our study also provides new information to the general literature that these teens could also be at risk for higher BMIs, as they also experienced poorer diets than those in parent-headed households.

The third hypothesis regarding food security was partially supported. Adolescents living in multi-generational homes reported more food insecurity in the form of going to school or bed hungry than those living in homes headed by parents. However, there were no significant differences from those living with only grandparents. Additionally, there were no significant differences by caregiver type for adolescents’ eating breakfast on weekends and no post hoc group differences in eating breakfast on weekdays. The higher rates of skipping meals for multi-generational homes may be due to the greater reliance on resource-sharing that resulted in the formation of these multi-caregiver families. Prior research has shown that low-income families are more likely to combine households as a strategy to increase resources [39].

A primary strength of this study is that it is the first to quantify and compare the nutritional intake of households that are headed by parents to those headed by grandparents and those that are multi-generational. Other strengths of the study include its nationally representative sample and adequate representation of adolescents from racial and financial minorities.

The data in this survey were based on self-reports from adolescents and, thus, limited to their recall. The researchers were limited to the methods of the dataset and operationalization of the variables. Although it would be possible to group dependent variables into summed categories of “nutrition” and “hunger frequency”, the research team chose to analyze each question separately due to the dearth of knowledge regarding the specific nutritional habits of children raised by their grandparents and in multi-generational homes. In addition, though this was a large study, while controlling for variables, the smaller sample size of the caregiving grandparents may have limited the results.

Mental health and stress are becoming more recognized factors in children’s lives but were not available for analysis in this dataset. Stress and conflict within households could be confounding variables related to higher BMI [25]. While the children have been living with their grandparents, we do not know how long they have been staying with them. Grandparents who have more time and experience with their grandchildren may have different attitudes towards child nutrition than those who have temporary custody [18].

Future studies are encouraged to utilize more complete measures of nutritional intake and exercise frequency to examine these relationships among children raised by different caregivers including those who prepare the family meals. Additionally, although the HBSC assesses the frequency of participants going to school or bed hungry when there is not enough food at home, it does not inquire further into food insecurity. Future studies are encouraged to examine this context in more depth. Though there were no significant group differences by caregiver type regarding affluence in the current sample of adolescents, future studies should consider examining these relations among high-risk, low-income groups, as they may be at further risk of food insecurity and its resulting consequences. Additionally, to maximize statistical power, the “Parents-Only” and “Grandparents-Only” groups included households in which either one or two parental/grandparental caregivers were present. Future studies are encouraged to examine the effects of living in a household with only one versus two or more caregivers, as this may affect the availability of shared resources.

## 5. Conclusions

Adolescents living with grandparents are at risk for poorer dietary behaviors, food insecurity as well as higher BMI. Our finding that living in a multi-generational home may be associated with higher food insecurity and places children at a higher risk of poor diets and obesity fits with and expands the previous literature on the topic. Our results highlight the crucial need for grandparent caregivers to be recruited and offered nutrition education and intergenerational exercise promotion programs.

## Figures and Tables

**Table 1:** Descriptive statistics of measures.

Variable Name	Mean	SD	Missing (%)
Affluence (SES)	5.92	1.96	151 (1.2)
Breakfast Weekdays	4.32	1.98	135 (1.1)
Breakfast Weekends	2.65	0.61	478 (3.9)
Fruit	4.95	1.72	553 (4.5)
Vegetables	4.55	1.82	725 (6.0)
Sweets	4.07	1.77	754 (6.2)
Soft Drinks	4.14	1.98	628 (5.2)
Snacking with TV	3.78	1.64	3804 (31.2)
Snacking Computer/Video Games	3.13	1.78	3873 (31.8)
Fast Food	3.90	1.48	141 (1.2)
Hunger/Food security	3.65	0.64	208 (1.7)
Computed BMI-for-Age Weight Status Category	2.42	0.78	2751 (21.8)

## Data Availability

This dataset is available to researchers at http://www.hbsc.org (accessed on 29 April 2022).
